# [Retracted] microRNA‑363‑3p inhibits cell growth and invasion of non‑small cell lung cancer by targeting HMGA2

**DOI:** 10.3892/mmr.2023.13034

**Published:** 2023-06-19

**Authors:** Chuanfu Jiang, Yang Cao, Ting Lei, Yu Wang, Junfeng Fu, Ze Wang, Zhenyang Lv

Mol Med Rep 17: 2712–2718, 2018; DOI: 10.3892/mmr.2017.8131

Following the publication of this paper, it was drawn to the Editor's attention by a concerned reader that certain of the data shown for the Transwell cell migration and invasion assays in Figs. 2C and 4C were strikingly similar to data appearing in different form in another article by different authors at a different research institution. Owing to the fact that the contentious data in the above article were under consideration for publication elsewhere at a similar time to when it was submitted to *Molecular Medicine Reports*, the Editor has decided that this paper should be retracted from the Journal. The authors were asked for an explanation to account for these concerns, but the Editorial Office did not receive any reply. The Editor apologizes to the readership for any inconvenience caused.

